# Reciprocal Regulatory Interaction between TRPV1 and Kinin B1 Receptor in a Rat Neuropathic Pain Model

**DOI:** 10.3390/ijms21030821

**Published:** 2020-01-27

**Authors:** Veronica Cernit, Jacques Sénécal, Rahmeh Othman, Réjean Couture

**Affiliations:** Department of Pharmacology and Physiology, Faculty of Medicine, Université de Montréal, Montréal, QC H3C 3J7, Canada; veronica.cernit@umontreal.ca (V.C.); jacques.senecal@umontreal.ca (J.S.); Rahmeh.othman@umontreal.ca (R.O.)

**Keywords:** bradykinin, B1 receptor, TRPV1, cytokines, hyperalgesia, allodynia, spinal cord, dorsal root ganglion, astrocytes, sensory fibers

## Abstract

Kinins are mediators of pain and inflammation and evidence suggests that the inducible kinin B1 receptor (B1R) is involved in neuropathic pain (NP). This study investigates whether B1R and TRPV1 are colocalized on nociceptors and/or astrocytes to enable regulatory interaction either directly or through the cytokine pathway (IL-1β, TNF-α) in NP. Sprague Dawley rats were subjected to unilateral partial sciatic nerve ligation (PSNL) and treated from 14 to 21 days post-PSNL with antagonists of B1R (SSR240612, 10 mg·kg^−1^, i.p.) or TRPV1 (SB366791, 1 mg·kg^−1^, i.p.). The impact of these treatments was assessed on nociceptive behavior and mRNA expression of B1R, TRPV1, TNF-α, and IL-1β. Localization on primary sensory fibers, astrocytes, and microglia was determined by immunofluorescence in the lumbar spinal cord and dorsal root ganglion (DRG). Both antagonists suppressed PSNL-induced thermal hyperalgesia, but only SB366791 blunted mechanical and cold allodynia. SSR240612 reversed PSNL-induced enhanced protein and mRNA expression of B1R and TRPV1 mRNA levels in spinal cord while SB366791 further increased B1R mRNA/protein expression. B1R and TRPV1 were found in non-peptide sensory fibers and astrocytes, and colocalized in the spinal dorsal horn and DRG, notably with IL-1β on astrocytes. IL-1β mRNA further increased under B1R or TRPV1 antagonism. Data suggest that B1R and TRPV1 contribute to thermal hyperalgesia and play a distinctive role in allodynia associated with NP. Close interaction and reciprocal regulatory mechanism are suggested between B1R and TRPV1 on astrocytes and nociceptors in NP.

## 1. Introduction

Neuropathic pain (NP) prevalence in the general population is estimated between 6.9% and 10% and remains a clinical problem without satisfactory treatment [1]. The identification of the underlying mechanisms and the development of novel pharmacological targets are prerequisite to treat NP-evoked spontaneous pain, hyperalgesia, and allodynia [2]. The kinin family including bradykinin (BK) and kallidin (Lys-BK) are mediators of pain and inflammation, which represent relevant therapeutic targets through their G-protein-coupled B1 and B2 receptors (B1R and B2R) [3,4]. Both receptors promote mechanical and thermal hyperalgesia [5,6]. Kinin B1R and/or B2R antagonists displayed antinociceptive effects in NP models induced by sciatic nerve injury [5,7,8,9], brachial plexus avulsion [10], type 1, and type 2 diabetes [6,11,12] and chemotherapy [13]. It is believed that B2R activation entails the onset of acute phase of inflammatory pain, while B1R is involved in the late phase of inflammatory hyperalgesia and NP [7,14,15]. This is consistent with the constitutive (with rapid internalisation and desensitisation) and inducible (with weak internalisation and desensitization) features of B2R and B1R, respectively [16].

Transient receptor potential vanilloid 1 (TRPV1), a noxious heat detector and integrator of thermal and chemical noxious stimuli, can be sensitized through direct phosphorylation in the presence of a variety of inflammatory mediators [17], including bradykinin (B2R) through intracellular PLC and PKC signaling [18,19]. Such interaction between TRPV1 and B1R has not been reported despite B1R was claimed to be expressed on primary sensory neurons [20,21] and thermal hyperalgesia was associated with enhanced spinal cord B1R and TRPV1 mRNA levels following spinal cord injury [22] and diabetic NP [6]. Thermal hyperalgesia associated with inflammation and tissue injury was impaired in TRPV1 knockout mice [23]. Likewise, B1R knockout mice show reduced thermal hyperalgesia and mechanical allodynia in NP models induced by sciatic nerve injury [24] and type 1 diabetes [25]. Moreover, B1R knockout mice dampened nociceptive responses to thermal noxious stimuli and capsaicin-induced TRPV1 activation [26]. Finally, activation of TRPV1 by capsaicin enhanced the expression of B1R in the spinal cord and caused hyperalgesia upon B1R activation [27]. Collectively, these findings suggest an interaction between B1R and TRPV1 in NP.

The stimulation of TRPV1 can enhance the production of pro-inflammatory cytokines such as TNF-α and IL-1β [28] that are known to enhance the expression of B1R through the activation of p-38 MAPK and NF-κB [29]. IL-1β also induces p-38 dependent sensitization of TRPV1-positive nociceptors [30] and TNF-α sensitizes and up-regulates TRPV1 expression on sensory neurons [31], contributing to enhanced mechanical and thermal hyperalgesia. IL-1β promotes astrocyte proliferation and activation through IL-1R signaling [32]. Activated astrocytes, in turn, release inflammatory mediators such as prostaglandins, chemokines, cytokines and nitrogen species that enhance hyperalgesia [33] contributing to NP. The upregulation of B1R in spinal dorsal horn microglia by pro-inflammatory cytokines has recently been shown as an early mechanism in diabetic NP [15] that is congruent with the role of microglia in NP [34].

Hence, interaction between B1R and TRPV1 appears as a potential NP mechanism. This hypothesis was addressed using the classical model of NP induced by unilateral partial sciatic nerve ligation (PSNL). The impact of one-week blockade of B1R or TRPV1 was determined on: (I) behavioral nociceptive responses (thermal hyperalgesia and allodynia); (II) B1R and TRPV1 gene expression together with the expression profile of TNF-α and IL-1β in the lumbar spinal cord and dorsal root ganglion (DRG). The cellular localization of B1R and TRPV1 was investigated on primary sensory neurons, astrocytes, and microglia.

## 2. Results

### 2.1. Changes of Nociceptive Behavior after PSNL

**Figure 1:**
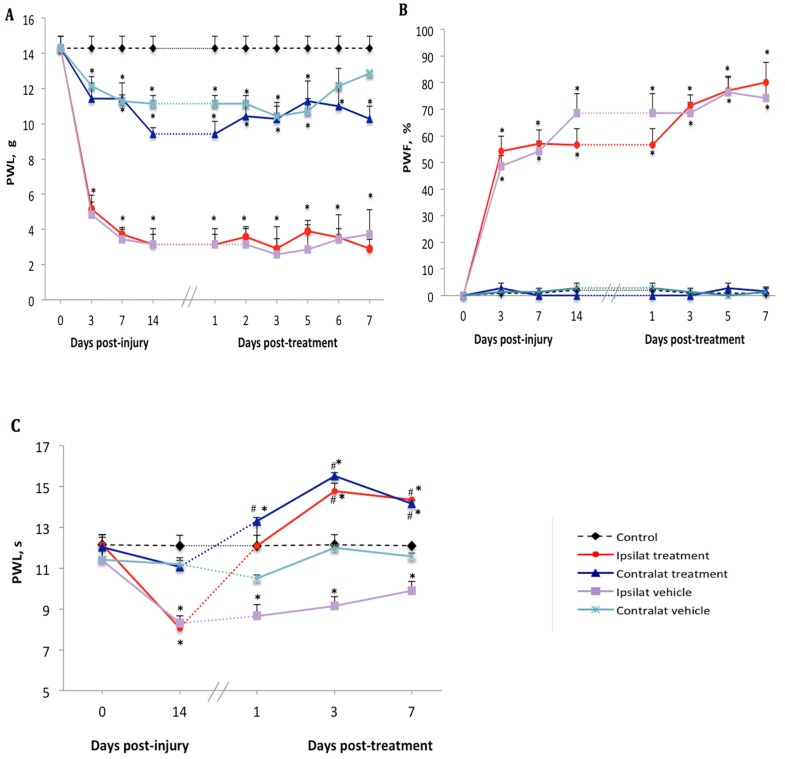
Nociceptive behavior after treatment with kinin B1 receptor (B1R) antagonist. Effect of one-week daily administration of SSR240612 (10 mg·kg^−1^, i.p.) and its vehicle on mechanical allodynia (**A**), cold allodynia (**B**) and heat-hyperalgesia (**C**). Post-treatment values from days 1 to 7 are the average of the effect obtained during the first 2 h post-administration of the antagonist. Data represent the mean ± SEM of control (*n* = 7), vehicle-treated (*n* = 7) and SSR240612-treated (*n* = 8) groups. At some points, SEM are not visible because they overlap with the points. Control values are from the ipsilateral sham-operated side of untreated rats. Statistical analysis indicates * *p* < 0.05 vs. control and ^#^ *p* < 0.05 vs. vehicle-treated group, (F = 4.66, *p* < 0.001), for all three types of hypersensitivity.

There was no difference in baseline values of nociceptive reflexes to noxious (mechanical or thermal) stimulation between the hind paws in any groups of rats (Figure 1A–C). On day 3 after PSNL, a significant decrease (from 14.3 ± 1.4 to 5.1 ± 0.8 g) in paw withdrawal latency (PWL) to mechanical stimuli was seen on the ipsilateral paw. Although less striking, PWL on the contralateral paw was also significantly reduced (from 14.3 ± 1.4 to 11.4 ± 1.8 g) compared to control (Figure 1A). Paw withdrawal frequency (PWF) to acetone-evoked cold allodynia was significantly increased on the ipsilateral paw (from 0 to 54.3 ± 11.2%) but remained unchanged on the contralateral paw (from 0 to 2.8 ± 3.6%) compared to control (Figure 1B). Thermal hyperalgesia did not occur on days 3 and 7 after PSNL, yet on day 14 a significant decrease (from 11.8 ± 0.9 to 8.1 ± 0.5 s) in PWL to heat stimuli was observed on the ipsilateral paw compared to control and to the contralateral paw where the PWL change was not significant (from 12.1 ± 1.1 to 11.1 ± 0.6 s) (Figure 1C).

### 2.2. Effect of the B1R Antagonist on PSNL-Induced Nociceptive Behavior

Beginning on day 14 post-PSNL, daily treatment for 7 days with the B1R antagonist SSR240612 (10 mg·kg^−1^, i.p.) did not reverse mechanical hypersensitivity in PSNL rats in both ipsilateral (2.9 ± 1.2 g) and contralateral (10.2 ± 1.8 g) paws when compared to vehicle values (3.7 ± 1.1 and 12.8 ± 1.4 g, respectively) (Figure 1A). Treatment with SSR240612 also failed to affect the time-course of PSNL-induced cold allodynia in both ipsilateral (80.0 ± 12.1%) and contralateral (0%) paws on day 7 post-treatment when compared to the vehicle (Figure 1B). In contrast, daily administration of the B1R antagonist had an inhibitory effect on thermal hyperalgesia compared to the vehicle. A significant augmentation of PWL occurred on the ipsilateral (from 8.1 ± 0.6 s prior to treatment to 14.2 ± 1.0 s on day 7 post-treatment) and contralateral (from 11.1 ± 0.8 to 14.7 ± 1.0 s) paws when compared to the vehicle-treated group (Figure 1C). Inhibition of heat hyperalgesia by the B1R antagonist was more efficient from day 3 to day 7 post-treatment.

### 2.3. Effect of the TRPV1 Antagonist on PSNL-Induced Nociceptive Behavior

**Figure 2:**
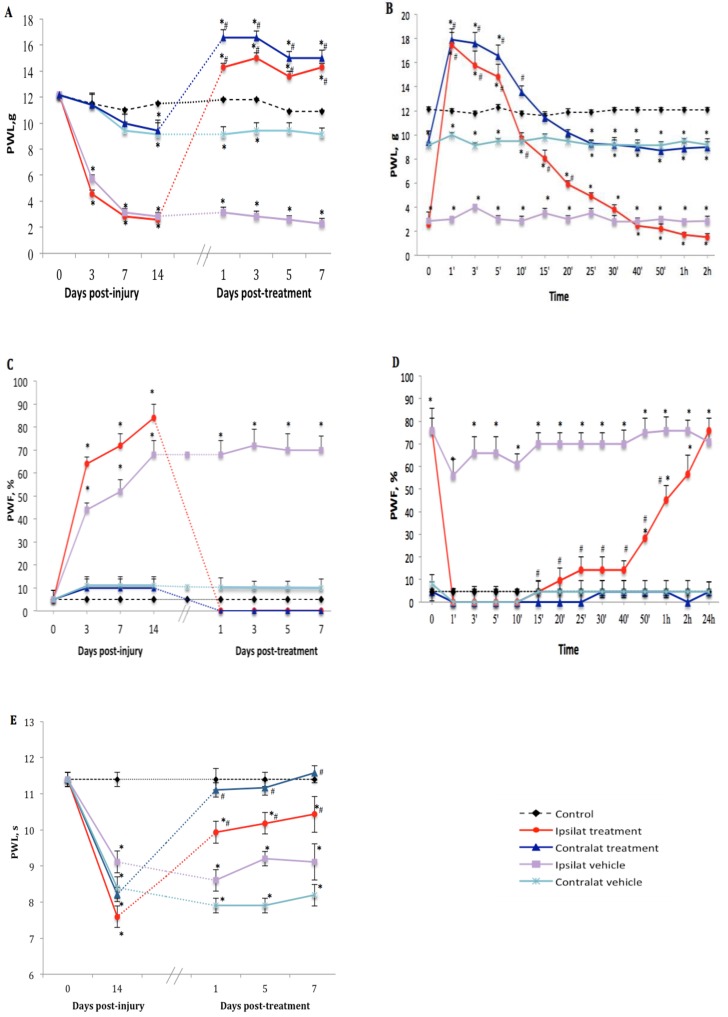
Nociceptive behavior after treatment with transient receptor potential vanilloid 1 (TRPV1) antagonist. Effect of one-week daily administration of SB366791 (1 mg·kg^−1^, i.p.) and its vehicle on mechanical allodynia (**A**,**B**), cold allodynia (**C**,**D**), and heat-hyperalgesia (**E**). Time-course inhibitory effects of SB366791 on allodynia are given in B and D. Post-treatment values from days 1 to 7 in A and C represent the average of peak inhibition during the first 5–10 min post-administration. In E, PWL was still completely inhibited at 24 h post-treatment and therefore, the effect of the antagonist was similar before and after administration. Thus, time-course effect is not shown in E. Data represent the mean ± SEM of control (*n* = 7), vehicle-treated (*n* = 7) and SB366791-treated (*n* = 8) groups. At some points, SEM are not visible because they overlap with the points. Control values are from the ipsilateral sham-operated side of untreated rats. Statistical comparison to control is indicated by * *p* < 0.05, (F = 5.98, *p* <0.001), and to vehicle by ^#^ *p* < 0.05, (F = 5.98, *p* < 0.001), for all three types of hypersensitivity.

The TRPV1 antagonist SB366791 (1 mg·kg^−1^, i.p.) inhibited nociceptive behavior manifested by tactile allodynia in PSNL rats in comparison to vehicle-treated group. Thus one-week treatment with the antagonist inhibited PWL on both ipsilateral and contralateral paws from 2.6 ± 0.7 to 14.3 ± 1.4 g and from 9.4 ± 0.8 to 16.6 ± 3.1 g, respectively (Figure 2A). We noticed that TRPV1 antagonist reversed and blocked mechanical hypersensitivity for up to 40 min with maximum effect at 1 min post-drug administration (17.5 ± 0.6 and 17.9 ± 0.6 g on ipsi- and contralateral paws, respectively) exceeding the cut-off value of PWL estimated at 15 g. This inhibitory and analgesic effect returned gradually to baseline values within 25 min (ipsilateral) and 15 min (contralateral) after SB366791 administration (Figure 2B). Similar inhibition was observed from day 1 to day 7 post-treatment.

Cold allodynia occurred only on the ipsilateral paw after PSNL. Daily treatment with SB366791 abolished PWF from 83.6 ± 12.7 to 0% (*p* < 0.05) on the ipsilateral paw from day 1 to day 7 post-treatment. The antagonist had no significant effect on the contralateral paw (Figure 2C). The inhibitory effect of SB366791 on cold allodynia lasted about 40 min (*p* < 0.05) and returned gradually to around 60% of PWF during the next 2 h to reach ipsilateral vehicle values on the next day after treatment (Figure 2D).

SB366791 reversed thermal hyperalgesia on the ipsilateral (from 7.6 ± 0.7 to 10.4 ± 1.6 s) and contralateral (from 8.2 ± 1.3 to 11.6 ± 1.8 s) paws at day 7 post-treatment, yet the inhibition was somewhat similar at 1, 5 and 7 days post-treatment (Figure 2E). Time-course inhibition was not shown herein because thermal hyperalgesia remained inhibited from 10 min to 24 h post-SB366791 at the time of the next day treatment.

The analgesic/hypoalgesic effect of SSR240612 on thermal nociception and SB366791 on mechanical sensitivity has not been investigated in this study but could be due to a spinal/supraspinal mechanism as non-peptide antagonists are known to pass the blood–brain barrier. This is supported by the finding that the administration of a peptide B1R antagonist (R-954), which does not pass blood–brain barrier, failed to display analgesic/hypoalgesic effect in the same model of neuropathic pain [35]. Further studies are warranty to support this hypothesis and to identify the putative target in the CNS for SSR240612 and SB366791.

### 2.4. Effect of B1R and TRPV1 Antagonists on Spinal Cord mRNA Levels

**Figure 3:**
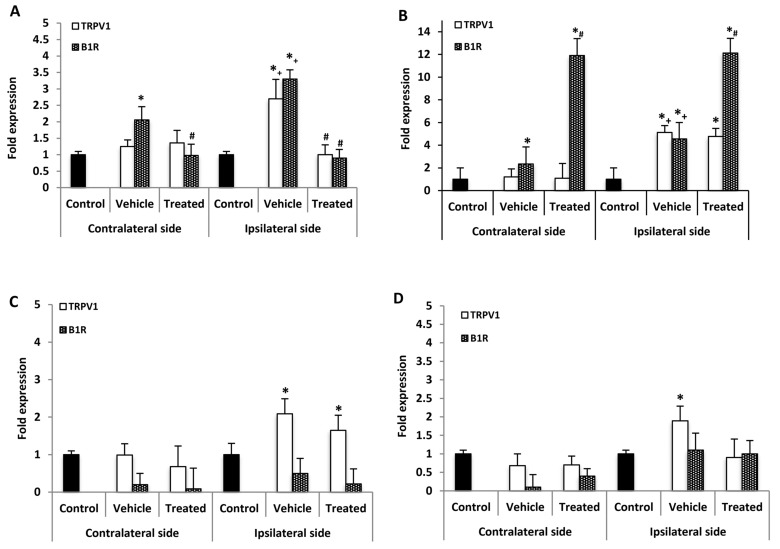
mRNA levels of B1R and TRPV1 in the spinal cord and dorsal root ganglion after treatment with SSR240612 and SB366791. mRNA levels of TRPV1 (white) and B1R (hatched) are shown in the contralateral and ipsilateral spinal cord (**A**,**B**) and dorsal root ganglion (**C**,**D**) of PSNL rats. Shown are the effects of one-week daily treatment with SSR240612 (10 mg·kg^−1^, **A**,**C**) and SB366791 (1.0 mg·kg^−1^, **B**,**D**). Values are fold-change ± SEM of control (*n* = 7), vehicle-treated (*n* = 7), SB366791-treated (*n* = 8) and SSR240612-treated (*n* = 8) groups. Statistical significance is indicated by * *p* < 0.05, (F = 4.66, *p* < 0.001) vs. control; ^#^ *p* < 0.05, (F = 4.66, *p* = 0.01) vs. vehicle; and + *p* < 0.05, (F = 4.66, *p* < 0.001) ipsilateral vehicle vs. contralateral vehicle. Each gene was compared to its own control for statistical analysis, yet only one control (black) is depicted in graphs (fold = 1).

B1R and TRPV1 mRNA levels were measured in the lumbar spinal cord (SC) 21 days post-PSNL, 3 h after the last antagonist administration. B1R mRNA but not TRPV1 mRNA was significantly increased in the contralateral SC compared with control. However, both B1R and TRPV1 mRNA levels were significantly increased on the ipsilateral SC when compared to the contralateral or control SC. One-week treatment with SSR240612 reversed completely the enhanced expression of B1R and TRPV1 on the ipsilateral and that of B1R on the contralateral SC compared to vehicle (Figure 3A). In contrast, SB366791 enhanced by 12-fold B1R mRNA level in both sides of the SC in PSNL rats when compared to vehicle (Figure 3B). SB366791 treatment had no significant effect on TRPV1 mRNA level on both sides of the SC in comparison to vehicle (Figure 3B).

PSNL increased significantly TNF-α but not IL-1 β mRNA levels with respect to control, on both contralateral and ipsilateral SC (Table 1). The mRNA levels of each cytokine were not significantly different between the contralateral and ipsilateral SC. SSR240612 enhanced significantly IL-1β and TNF-α mRNA expression on both sides of the SC with respect to vehicle. The TRPV1 antagonist failed to affect TNF-α mRNA levels on both sides but enhanced significantly IL-1β mRNA levels on both sides of the SC compared to vehicle (Table 1).

### 2.5. Effect of B1R and TRPV1 Antagonists on mRNA Levels in DRG

No significant change of B1R mRNA expression level was detected in the contralateral and ipsilateral DRG of PSNL rats in comparison to control (Figure 3C). One-week treatment with SSR240612 failed to affect B1R mRNA levels in both DRG sides in comparison to vehicle (Figure 3C). Whereas TRPV1 mRNA level was not affected in contralateral DRG of PSNL rats, it was increased on the ipsilateral DRG in comparison to control; treatment with SSR240612 had no significant effect on TRPV1 expression on the ipsilateral DRG (Figure 3C). The enhanced TRPV1 mRNA expression on the ipsilateral DRG was no longer significant in PSNL rats treated with SB366791 (Figure 3D). PSNL enhanced by 3.9-fold the expression of TNF-α on the ipsilateral DRG while changes were not significant on the contralateral DRG (Table 1). One-week treatment with SSR240612 or SB366791 did not significantly affect the expression of TNF-α compared with vehicle on both sides (Table 1). The expression of IL-1β was not significantly affected by PSNL or by both antagonists on the contralateral and ipsilateral DRG (Table 1).

### 2.6. B1R Protein Expression in the Ipsilateral Dorsal Horn (iDH)

**Figure 4:**
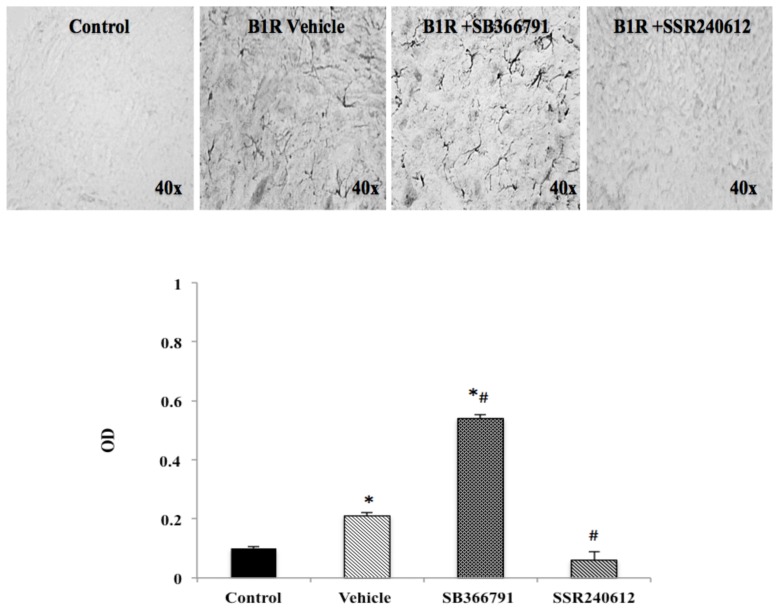
B1R protein expression in the ipsilateral dorsal horn of the spinal cord. Images represent digital microphotographs of B1R protein expression in the ipsilateral dorsal horn of partial sciatic nerve ligation (PSNL) rats revealed by color-based (DAB chromogen) immuno-enzymatic (biotin-streptavidin HRP) technique. Quantitative optical density values (on a scale of 1.0) are given for B1R in control, vehicle-treated, SB366791 and SSR240612-treated rats. Values are the mean ± SEM of 5 rats per group (each value is the mean of 5 sections per rat). Significance is indicated by * *p* < 0.05 vs. control and ^#^ *p* < 0.05 vs. vehicle-treated group, (F = 3.95, *p* < 0.0001). Magnification is 40×.

PSNL caused widespread distribution of B1R immunostaining in the ipsilateral dorsal horn of the lumbar spinal cord. PSNL increased by more than two-fold B1R protein expression compared to control, and this increase was significantly inhibited to control values by SSR240612 treatment (Figure 4). In contrast, treatment with the TRPV1 antagonist, SB366791, enhanced by close to 6-fold B1R immunostaining in the ipsilateral dorsal horn and the increase was significant compared to control and vehicle-treated group (Figure 4).

### 2.7. Localization of B1R and TRPV1 in the Ipsilateral Dorsal Horn (iDH) and DRG (iDRG)

**Figure 5:**
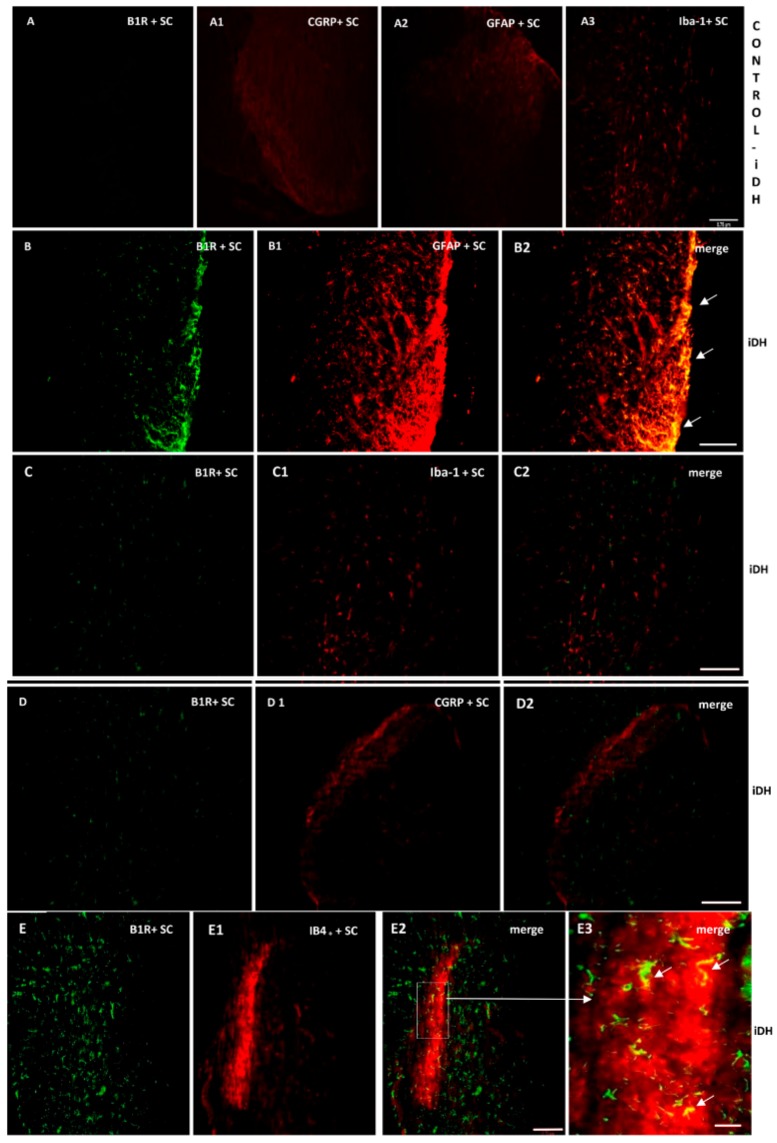
Cellular distribution of B1R in the ipsilateral Dorsal Horn (iDH). Representative microphotographs by immunofluorescence of B1R (green, **B**–**E**) relative to GFAP (red, **B1**), Iba-1 (red, **C1**), CGRP (red, **D1**) and IB4+ (red, **E1**) in the spinal cord (SC) of PSNL rats. Control spinal cord for B1R (A) expression exhibits very weak-to-no signal. Expression of CGRP (**A1**), GFAP (**A2**) and Iba-1 (**A3**) is moderate in the spinal cord of control animals. B1R immunolabelling is colocalized with astrocytes (arrows, GFAP, **B2**) and non-peptide fibers (arrows, IB4+, **E2**, **E3**), but not with microglia (Iba-1, **C2**) and peptide fibers (CGRP, **D2**) in the spinal dorsal horn of PSNL rats. E2 is enlarged in E3 as indicated by the white square and arrow. Scale bar = 120 µm (all panels, 10×), except 60 µm (**E3**, 20×).

**Figure 6:**
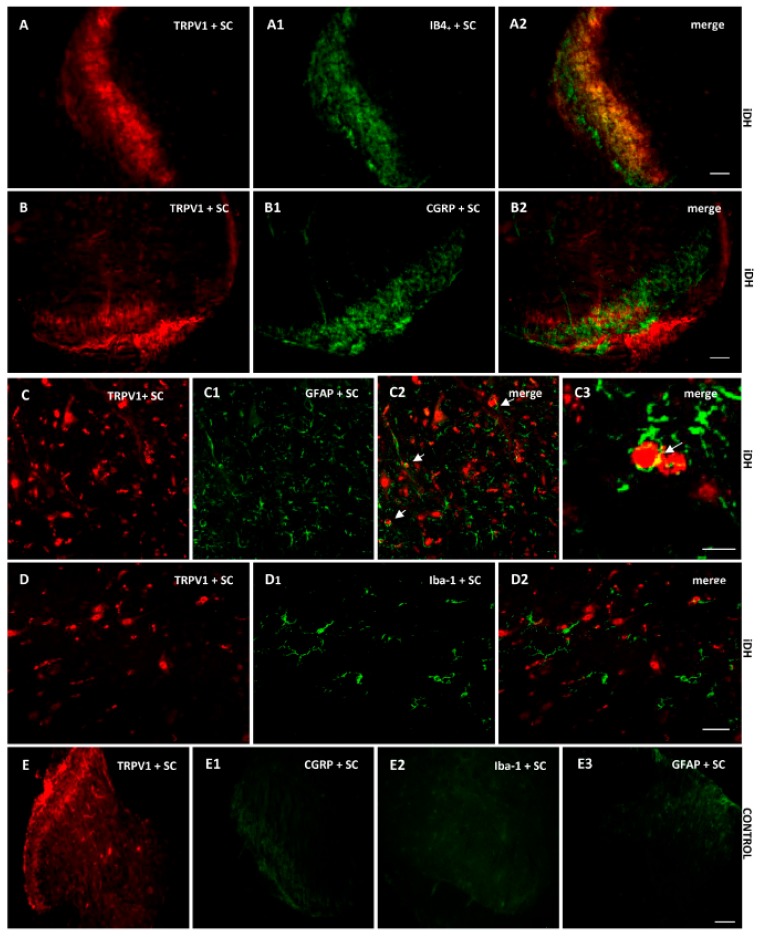
Cellular distribution of TRPV1 in the ipsilateral Dorsal Horn (iDH). Representative microphotographs by immunofluorescence of TRPV1 (red, **A**–**D**) relative to IB4+ (green, **A1**), CGRP (green, **B1**) GFAP (green, **C1**), Iba-1 (green, **D1**) in the spinal cord (SC) of PSNL rats. TRPV1 colocalizes weakly with peptide (CGRP, **B2**), and more abundantly with non-peptide fibers (IB4+, **A2**) and astroglia (GFAP, **C2 C3**), but not with microglia (**D2**). The same markers are shown in iDH of control rats. Scale bar = 120 µm (all panels, 10×), 60 µm (**C**–**C2** and **D**–**D2**, 20×), 75 µm (**C3**, 60×).

As B1R immunostaining seems to occur on astrocytes depicting large and ramified processes after PSNL (Figure 4), the co-expression of B1R and TRPV1 on astrocytes was further studied by immunofluorescence. Data confirm the presence of B1R on astrocytes labelled with GFAP in the SC of PSNL rats (Figure 5B2, merged as yellow). In contrast, B1R did not colocalize with Iba-1 on microglia (Figure 5C2) or on peptide sensory fibers labelled with CGRP (Figure 5D2). Nevertheless, B1R was detected on non-peptide sensory fibers labelled with IB4+ in the SC of PSNL rats (Figure 5(E2,E3)). Likewise, Figure 6 shows the colocalization of TRPV1 on non-peptide sensory fibers labelled with IB4+ (Figure 6A2, merged as yellow) and weakly on peptide sensory fibers labelled with CGRP (Figure 6B2). Noteworthy, TRPV1 was also present on astrocytes labelled with GFAP (Figure 6(C2,C3)), but not on microglia labelled with Iba-1 (Figure 6D2). Hence, B1R immunofluorescence was virtually absent in control dorsal horn (Figure 5A), but markedly enhanced after PSNL (Figure 5B–E). While GFAP immunofluorescence was detectable in control dorsal horn (Figure 5A2), the intensity of GFAP (Figure 5B1) was also enhanced in PSNL rats relative to control.

**Figure 7:**
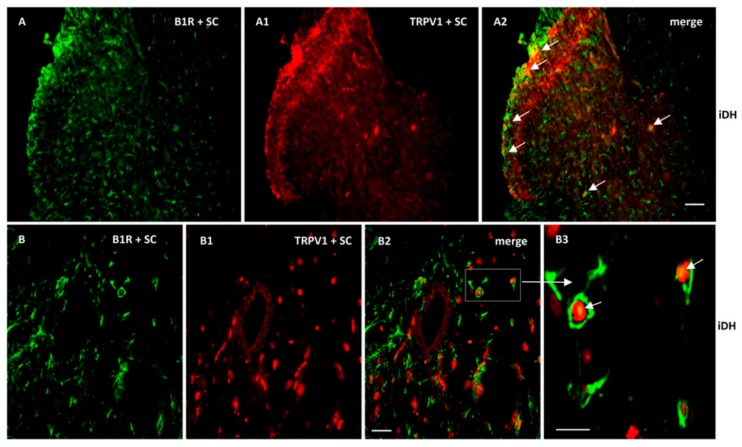
Co-expression of B1R and TRPV1 in the ipsilateral Dorsal Horn (iDH). Representative microphotographs by immunofluorescence of B1R (green, **A**,**B**) and TRPV1 (red, **A1**,**B1**) in the spinal cord (SC) of PSNL rats. Images **A2**, **B2** and **B3** reveal a co-expression of B1R and TRPV1 (white arrows) at superficial layers of the dorsal spinal cord. Scale bar = 120 µm (**A**–**A2**, 10×), 60 µm (**B**–**B2**, 20×) and 50 µm (**B3**, 40×).

**Figure 8:**
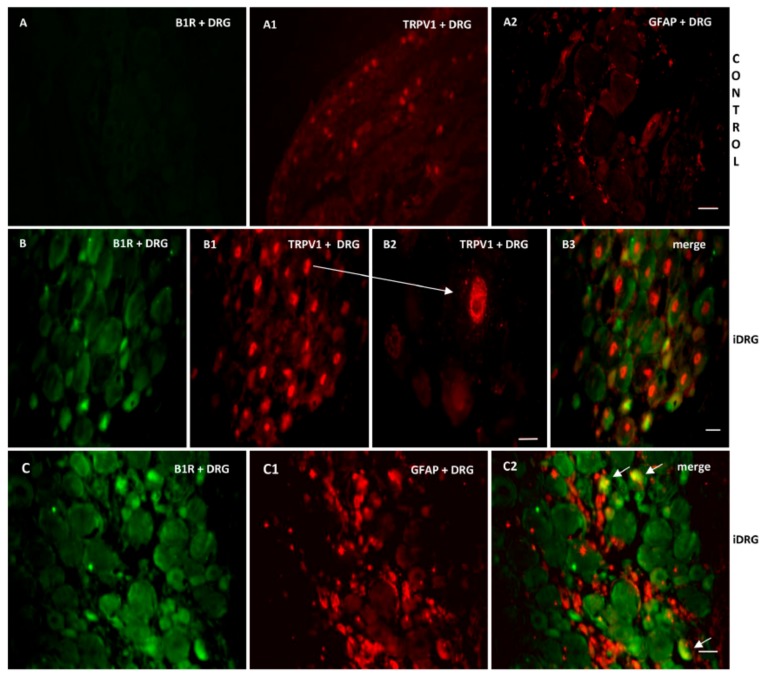
Co-expression of B1R with TRPV1 or GFAP in the ipsilateral lumbar DRG (iDRG). Representative microphotographs by immunofluorescence of B1R (green, **B**,**C**) and TRPV1 (red, **B1**,**B2**) and GFAP (red,**C1**) in the iDRG of PSNL rats. Control iDRG (**A**,**A1**,**A2**) exhibit no staining for B1R (**A**) and a moderate staining for both TRPV1 (**A1**) and GFAP (**A2**). B1R and TRPV1 are co-expressed in the DRG (**B3**). Enlarged DRG showing TRPV1 staining around the nucleus (arrow, B2). A punctual co-localization of B1R with GFAP (arrow, possibly issued from satellite cells) was found in the lumbar DRG (**C2**). Scale bar = 120 µm (**A**–**A2**, 10×), 60 µm (other panels, 20×), 75 µm (**B2**, 60×).

As B1R and TRPV1 immunofluorescence were present on similar structures in the SC of PSNL rats, we further studied their possible colocalization in the SC and DRG. Thus, we found co-expression of B1R and TRPV1 in the superficial layers of the dorsal spinal cord (Figure 7(A2, B2, B3)). Despite the weak B1R mRNA levels in DRG of PSNL rats, reliable B1R immunofluorescence was detected in the PSNL DRG (Figure 8B,C) in comparison to control DRG where B1R was not detectable (Figure 8A). This is consistent with the immunodetection of B1R on non-peptide sensory fibers in the SC (Figure 5(E2,E3)). The intensity of TRPV1 and GFAP immunofluorescence was also higher in the DRG of PSNL rats compared to control DRG (Figure 8). TRPV1 immunofluorescence was found in the cytoplasm around the nucleus (Figure 8B2) as for B1R (Figure 8B) where both were co-expressed in several DRG of PSNL rats (Figure 8B3). Noteworthy, B1R partially co-localized with GFAP (possibly issued from satellite cells) in the lumbar DRG of PSNL rats (Figure 8C2). 

**Figure 9:**
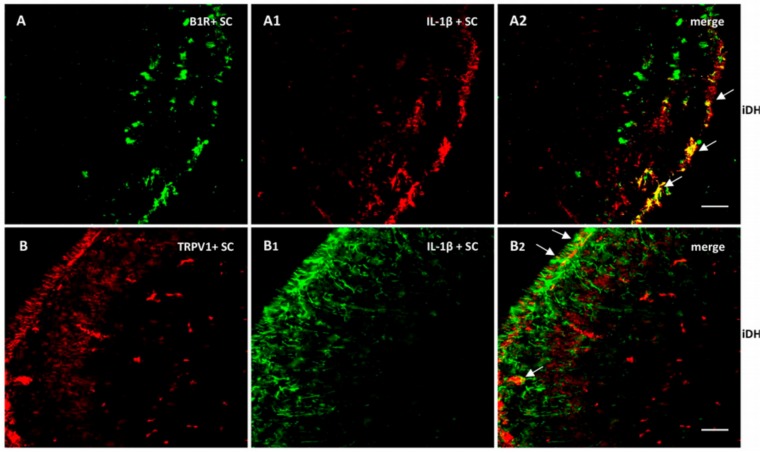
Distribution of B1R and TRPV1 in relation with IL-1β in the ipsilateral Dorsal Horn (iDH). Representative microphotographs by immunofluorescence of B1R (green, **A**) and IL-1β (red, **A1**) in the spinal cord of PSNL rats. Image **A2** shows colocalization between B1R and IL-1β (yellow spots). Likewise, TRPV1 (red, **B**) and IL-1β (green, **B1**) colocalize (**B2**) in the superficial layers of the spinal cord of PSNL rats. However, this colocalization is sparsely expressed. Scale bar = 120 µm. Magnification 10×.

As we found high levels of IL-1β mRNA in the spinal cord, we verified its expression and colocalization with B1R and TRPV1. As depicted in Figure 9, IL-1β largely colocalized with B1R (A2) and partially with TRPV1 (B2). Collectively, these findings suggest a possible interaction between B1R and TRPV1 in superficial laminae of the dorsal spinal cord and on sensory fibers in the PSNL rat.

## 3. Discussion

The main findings of this study performed in the rat model of PSNL are that:(1) B1R was involved in thermal hyperalgesia, but not in mechanical and cold allodynia; (2) TRPV1 was involved in thermal hyperalgesia and in mechanical and cold allodynia; (3) B1R and TRPV1 expressions (mRNA and protein) were enhanced in the spinal cord; (4) B1R mRNA and protein expressions were further increased by TRPV1 antagonism; (5) B1R antagonist reversed the upregulation of B1R (protein and mRNA) and TRPV1 (mRNA); (6) B1R and TRPV1 were immunodetected on astrocytes and non-peptide sensory fibres but not on microglia; (7) TRPV1 and B1R were colocalized in the spinal dorsal horn and DRG; (8) B1R was co-expressed with IL-1β, suggesting a role for this cytokine in the induction and upregulation of B1R; (9) IL-1β mRNA was increased by B1R and TRPV1 antagonism in the spinal cord, suggesting that IL-1β is located upstream to B1R and TRPV1; (10) enhanced TNF-α mRNA levels by PSNL was further increased by B1R antagonism; (11) colocalisation of B1R and TRPV1 in the spinal dorsal horn and DRG suggests possible interaction either on astrocytes and/or sensory fibers (nociceptors). 

### 3.1. B1R in Neuropathic Pain

B1R antagonist inhibited heat hyperalgesia for up to 24 h. However, it did not alter the mechanical and cold allodynia after PSNL. This finding supports previous data showing that B1R^−/−^ mice [4,24] or pharmacological B1R blockade [5,7,8] reduced thermal hyperalgesia in NP models. Likewise, B1R^−/−^ mice [25] and B1R antagonism [6,11] alleviated heat hyperalgesia in streptozotocin-diabetic rodents. Another report showed, however, that chronic hypersensitivity to heat associated to sciatic nerve injury is not altered in double B1R/B2R knockout mice [36]. The reason for this latter discrepancy is not known but could be due to adaptive compensatory mechanisms in kinin receptor knockout mice. 

We found no correlation between the degree of B1R expression in PSNL and nociceptive behavior elicited by mechanical or cold stimuli. Indeed, during the blockade and suppression of B1R expression by the B1R antagonist, allodynia remained untouched. The absence of B1R involvement in cold and mechanical allodynia after B1R blockade is consistent with previous studies after sciatic nerve injury [5,37] and with the development of nerve-injury-induced tactile hypersensitivity in B1R knockout mice [37]. Nonetheless, other studies showed the ability of B1R antagonism to reduce mechanical hyperalgesia or allodynia [8,24]. The reason for this discrepancy is unknown and might be related to differences in species or NP models. The type of antagonist might not be so critical as a similar dose and regimen of SSR240612 inhibited allodynia in diabetic rats [6,12]. 

PSNL-induced gene and protein overexpression of B1R in the spinal cord that is in line with previous autoradiographic studies showing upregulation of B1R binding sites in the ipsilateral and contralateral spinal cord at 2 and 14 days post-PSNL [5]. However, we did not detect B1R mRNA in lumbar DRG at 21 days post-PSNL, contrary to another study showing an increased level of B1R mRNA within the lumbar DRG at 14 days post-nerve injury [7]. Nevertheless, we found immunoprotein expression of B1R in non-peptide sensory fibers at the level of the spinal cord and DRG. One study reported newly expressed B1R in large diameter myelinated DRG neurons in PSNL mice. The authors argued that BK mediates nociception through B1R in myelinated neurons and ERK phosphorylation in nerve-injured mice [38]. 

In our study, B1R and IL-1β followed a similar pattern of expression in the spinal cord after PSNL. This is keeping with the role of IL-1β in the induction of B1R through the NF-κB pathway [4,39]. IL-1β is known to be responsible for thermal and mechanical hypersensitivity [40]. IL-1β receptors were found on activated astrocytes [41] from which IL-1β is produced [33]. Given that B1R was also found on activated astrocytes, and B1R and IL-1β were colocalized in the spinal cord after PSNL, one can suggest that B1R upregulation after PSNL is mediated by astrocyte-derived IL-1β. Such interaction could explain the upregulation of B1R (concomitantly with IL-1β) under TRPV1 antagonism and highlights a regulatory function for TRPV1 on B1R expression. The possibility that the compensatory upregulation of the pro-nociceptive B1R and IL-1β could diminish the therapeutic effect of TRPV1 antagonism in NP warranty further investigation.

B1R and TRPV1 are colocalized in the spinal cord and DRG and both are expressed in activated astrocytes and non-peptide sensory fibers in PSNL rats. Hence, our study supports the presence of TRPV1 on astrocytes [42]. In addition, activated astrocytes that express B1R were surrounding TRPV1-containing sensory fibers in the spinal cord dorsal horn (Figure 6C3 and Figure 7B3). Such physical proximity of the structures bearing B1R and TRPV1 emphasizes the possibility of mutual regulation between these two receptors. As B1R and TRPV1 were colocalised on astrocytes and sensory fibres, a direct physical interaction between them cannot be excluded as demonstrated for B2R which activates TRPV1 through PLC-PKCε-dependent phosphorylation [18,19]. The possibility that B1R can also activate TRPV1 through a similar phosphorylation mechanism remains to be investigated.

B1R protein expression was not detected in peptide (CGRP) sensory fibers after PSNL, which is consistent with the absence of B1R on primary sensory C-fibers labelled with CGRP in the pancreas of diabetic rat [43]. B1R was seen on a few microglia (Iba-1) only after PSNL and this distribution contrasts with the strong detection of B1R on astrocytes. Other experimental paradigms support B1R expression on microglia, notably in streptozotocin-induced diabetic neuropathy [6], LPS-induced inflammation [39] and in BK-induced microglia motility and chemotaxis in cultured cells [44]. The weak expression of B1R on microglia after 3 weeks of PSNL does not necessarily exclude a role for microglial B1R in the onset and late phase of NP.

### 3.2. TRPV1 in Neuropathic Pain

TRPV1 is commonly recognized as a main noxious heat sensor, being involved in thermal hyperalgesia [22,23,45], inflammatory pain [46], notably facial pain [47,48]. Our study suggests that TRPV1 could also be involved in mechanical and cold hypersensitivity during NP as shown for facial pain [47,48], yet its role appears shorter lasting than in heat hyperalgesia. The TRPV1 antagonist SB366791 dose-dependently alleviated heat and mechanical sensitivity in the model of chronic constriction injury of the sciatic nerve in mice when injected into the injured paw [49]. This is consistent with the rapid onset effect (within 1 min) of SB366791 to block pain behavior in our model and may indicate a peripheral site of action on nociceptors. This finding also suggests that the therapeutic value of TRPV1 blockade is superior on thermal hyperalgesia than on allodynia as the protective effect of SB366791 on heat hypersensitivity lasted up to one day post-treatment in PSNL rats.

We observed a similar pattern of TRPV1 and TNF-α mRNAs upregulation in ipsilateral spinal cord and DRG, which is consistent with the role of TNF-α in the upregulation of TRPV1 in sensory neurons [31] and in the trafficking of TRPV1 to the nociceptor membrane [50], which may contribute to mechanical and cold hypersensitivity mediated by TRPV1. However, because the B1R antagonist suppressed the upregulation of TRPV1 and increased the expression of IL-1β and TNF-α in the spinal cord of PSNL rats, it is suggested that B1R upregulates TRPV1 by a mechanism independent on IL-1β and TNF-α. Again, this suggests a reciprocal regulatory control between B1R and TRPV1, which warranty further investigation.

## 4. Materials and Methods

### 4.1. Animal Care Procedure

Experiments were carried out in 44 male Sprague Dawley (SD) rats (225–250 g) purchased from Charles River Laboratories (St-Constant, QC, Canada). Animals were housed two per cage and kept in a controlled environment and pathogen-free conditions (room temperature 23 °C, humidity 40%, 12/12h light/dark cycle). Standard chow diet (Charles River Rodent) and tap water were accessible *ad libitum*. Prior to experimentation, rats were allowed to acclimate for 5–7 days. Experiments were performed during the light phase of the cycle (9:00 AM–4:00 PM). The Animal Care Committee of the Université de Montréal approved all research procedures (Protocols 16–140, 17–140), which complied with the guiding principles for animal experimentation as enunciated by the Canadian Council on Animal Care.

### 4.2. Partial Sciatic Nerve Ligation (PSNL)

PSNL was performed according to the original description of Seltzer [51]. Under general anesthesia with 3% isoflurane (Santa Cruz Biotechnology, Mississauga, ON, Canada), animals were shaved at the dorsal part of the left thigh and the skin was treated twice with betadine (povidone-iodine, 5%, Purdue Frederick Co, Massillon, OH, USA) and once with alcohol 70% (Cole-Parmer Canada, QC, Canada). Under aseptic conditions and after incision of the skin at the upper-thigh level of the left paw, the sciatic nerve was exposed to the surface of the wound by slight upward movement of the *biceps femoris*. Then a tight ligation of the dorsal third to half thickness of the nerve was made with 6-0 silk thread, distal to the insertion of the posterior biceps semitendinosum muscle. After PSNL, the skin wound was sutured layer by layer with 4-0 silk thread to maintain the anatomical integrity of aponeurosis. The wound was then treated with 5% of betadine. Operated rats were allowed to recover from anesthesia in a warm, clean, dry and quiet environment. The wound healed within 1 to 3 days, and the rats behaved normally. There was no evidence of infiltration or redness in the wound area. 

Following surgery and over the next 2 days, the PSNL rats received the antibiotic Tribrissen 24%, 0.05 mL·kg^−1^, s.c., (Schering Canada Inc., Pointe Claire, QC, Canada) and the anti-inflammatory drug Carprofen 0.5 mg·kg^−1^, s.c., (Rimadyl 50 mg·mL^−1^, Zoetis Canada Inc., Kirkland, QC, Canada). Rats subjected to PSNL developed weakness of the hindpaw to the side of injury on the first day after nerve ligation. No paralysis of the operated paw was observed. All through the treatment, a protective but not self-mutilating behavior as autotomy was observed. No animals were excluded from the study.

### 4.3. Group Identification and Distribution of Animals

Rats were randomly divided into six groups as follows: two control groups (sham-operated, each group *n* = 7), two PSNL vehicle-treated group (each group *n* = 7), one PSNL B1R-antagonist treated group (*n* = 8) and one PSNL TRPV1-antagonist treated group (*n* = 8). Each antagonist treated group was compared to a separate control group and a separate vehicle-treated group. Such a group distribution was done based on preliminary data analysis of two pilot studies previously performed by our laboratory with the same protocol [35]. In the latter study, no statistical difference was found between ipsilateral and contralateral paw in sham-operated rats, excluding any effect of the surgery on allodynia and thermal hyperalgesia. On this basis, our results show data on ipsilateral sham-operated side, which is the control group throughout the present study. 

### 4.4. Behavioral Assessment

Nocifensive behavior was assessed on each hind paw (ipsilateral and contralateral to the injury) before and after surgery (on days 3, 7, 14, and 21 post-PSNL), and during one week of treatment (beginning from day 14 post-PSNL) with B1R and TRPV1 antagonists and their vehicles. Control groups (sham-operated rats) were tested in parallel.

### 4.5. Tactile Allodynia Test

Tactile allodynia (Foot Withdrawal Response to mechanical stimuli) was performed according to the method of Chaplan [52], by employing a calibrated series of 8 von Frey (VF) filaments from 0.7 to 26.0 g (Stoelting, Wood Dale, IL, USA). Prior to testing, rats were placed under a transparent plastic dome on a metal mesh support and were allowed for 20–30 min to acclimate. Starting from the lowest force filament, one at time, the VF filaments were applied perpendicular to the skin of hind-paw at mid-plantar region with adequate pressure until they bend. Five of ten rapid withdrawals (more than 50%) were measured as a positive response and the threshold in grams was noted. Absence of a response (less than 5 withdrawals) prompted the use of the next graded filament of increasing stiffness. As the nociceptive response to mechanical stimulation in control rats was set between 12–15 g, an upper cut-off for the paw withdrawal was estimated at 15 g. The measures were recorded at 1, 3 and 5 min after antagonist injection, then at 5–10 min interval for the first hour, and at 2 and 24 h post-injection.

### 4.6. Cold Allodynia Test

Cold allodynia (Foot Withdrawal Response to Acetone) was assessed as described by Choi [53]. Rats were placed under a transparent plastic dome on a metal mesh floor and after they acclimate, an acetone bubble formed at the tip of a plastic tube connected to a syringe was applied 5 times to the tested hind paw at the level of the heel. The measures were recorded at 5 min interval for the first hour after antagonist injection then at 2 and 24 h post-injection. Rats experiencing allodynia due to evaporation-evoked cooling respond with an abrupt withdrawal of tested paw to avoid pain. The frequency of foot withdrawal was calculated as a percentage (number of trials accompanied by brisk foot withdrawal) × 100/(number of total trials). 

### 4.7. Thermal Hyperalgesia Test

Thermal hyperalgesia (Paw Withdrawal Test) was assessed as described previously [54]. Paw withdrawal latency in seconds was measured by exposing the tested hind paw to a thermal stimulus. Briefly, rats were placed in Plexiglas cages on top of a glass floor to acclimate for a period of 30 min. The radiant thermal stimulus (UGO Basile Plantar Test 37370) was focused under the glass floor to project on the middle plantar surface of each hind paw of the rats. A cut-off time of the radiation beam was set up at 20 s to prevent tissue damage. The value of paw withdrawal was calculated as the average of three tests with the 3-min interval between each measurement. 

### 4.8. Pharmacological Treatments

B1R and TRPV1 antagonists were administered separately in different PSNL groups of rats to determine whether the blockade of B1R could affect the expression of TRPV1 and *vice-versa* if the blockade of TRPV1 could affect the expression of B1R. A high-affinity, non-peptide, selective bradykinin B1 antagonist SSR 240,612 [(2R)-2-[((3R)-3-(1,3-benzodioxol-5-yl)-3-[[(6-methoxy-2-naphthyl)-sulfonyl] amino] propanoyl)amino]-3-(4-[[2R,6S)-2,6 dimethylpiperidinyl]-methyl]phenyl)-*N*-isopropyl-*N*-methylpropanamide hydrochloride], which inhibited capsaicin-induced neurogenic inflammation [9], was kindly provided by Sanofi-Aventis R&D (Montpellier, France). A novel, potent, and selective, cinnamide TRPV1 antagonist SB366791, which caused a concentration-dependent inhibition of the pain response to noxious stimuli [55] was purchased from SynInnova Laboratories Inc. (Edmonton, AB, Canada). Both antagonists, being in the form of a powder, were dissolved in 5.0% solution of dimethyl sulfoxide (DMSO) obtained from Millipore Sigma (Oakville, ON, Canada).

The pharmacological treatments were initiated at 14-day post-PSNL, which is considered the peak time of neuropathic pain that remained stable up to 21-day post-PSNL. Antagonists of B1R (10 mg·kg^−1^) and TRPV1 (1 mg·kg^−1^) and their vehicles (5.0% DMSO) were given i.p. once daily at 9 AM. Doses of SSR240612 and SB366791 were based on previous studies in diabetic NP [6,12] and inflammatory heat hyperalgesia in a murine model of bone cancer pain [56].

### 4.9. Real-Time Quantitative Reverse Transcription Polymerase Chain Reaction (qRT-PCR)

Rats were sacrificed on day 21 after PSNL by decapitation under deep anesthesia with 3% isoflurane. Spinal cords were extracted and L3–L5 portions were harvested. The ipsi- and contralateral DRG of the same levels were excised after spinal laminectomy. Isolated tissues (spinal cord and DRG) were put immediately after sectioning into RNA*later* stabilization reagent (QIAGEN, Valencia, CA, USA) and stored at −80 °C until processed. Levels of B1R, TRPV1, TNF-α, and IL1-β mRNAs were measured using quantitative real-time PCR according to the method described previously [57]. Total RNAs were extracted from isolated tissues according to E.Z.N.A. HP Total RNA Kit Quick Guide instructions (Omega Bio-Tek, Norcross, GA, USA). First-strand cDNA synthesis was carried out from 200 ng total RNA with random hexamer primers and was used as template for each reaction with the All-in-One cDNA Synthesis SuperMix (Cedarlane, Burlington, ON, Canada). The following primer pairs were designed by Vector NTI software: 18S (X01117) forward: 5′-TCA ACT TTC GAT GGT AGT CGC CGT-3′ (363–386), reverse: 5′-TCC TTG GAT GTG GTA GCC GTT TCT-3′ (470–447); B1R (NM_030851) forward: 5′-GCA GCG CTT AAC CAT AGC GGA AAT-3′ (367–391), reverse: 5′-CCA GTT GAA ACG GTT CCC GAT GTT-3′ (478–454); IL-1β (NM_031512) forward: 5′-TGT CAC TCA TTG TGG CTG TGG AGA-3′ (247–270), reverse: 5′-TGG GAA CAT CAC ACA CTA GCA GGT-3′ (411–388); TRPV1 (NM_031982) forward: 5′-GCA CAA TGG GCA GAA TGA CAC CAT-3′ (575–598), reverse: 5′-GGC ATT GAC AAA CTG CTT CAG GCT-3′ (656–633), TNF-α (NM_012675) forward: 5′-ATG ATC CGA GAT GTG GAA CTG GCA-3′ (160–183), reverse: 5′-AAT GAG AAG AGG CTG AGG CAC AGA-3′ (257–234). Then qRT-PCR was performed with 300nM of each primer in the fluorescent double-stranded DNA binding dye SYBR Green Master Mix (Cedarlane, Burlington, ON, Canada). Fluorescent signal and the progress of DNA amplification was detected using the Mx3000p device (Stratagene, La Jolla, CA, USA). For standardization and quantification, rat 18S was amplified simultaneously. The qRT-PCT was run in triplicates. The relative fold-expression level of the target genes was calculated by the 2^−ΔΔ*C*T^ method.

### 4.10. Immunohistochemical and Immunofluorescence Procedures

Immunohistochemical and immunofluorescence procedures for protein analysis were processed as described previously [58]. Spinal cord sections and ipsi- and contralateral DRG of L3–L5 levels from control and nerve-injured rats were quickly removed from sacrificed rats. Collected tissues were immediately drop-fixed in ice-cold freshly made 4% paraformaldehyde (PFA) in a buffered solution and kept for 24 h at room temperature (RT). After fixation, tissues were transferred to 70% ethanol and stored at 4 °C overnight. The next day, collected tissues were embedded in paraffin wax and paraffin blocs were refrigerated, being kept at 4 °C until use. For immunofluorescent staining, paraffin blocs were serially cut into 8-μm thick coronal sections with a microtome and mounted onto glass slides. For further antibody staining, paraffin wax removal, rehydration, and antigen retrieval from samples were performed. Wax was removed by three changes immersion of slides in fresh xylene solution, 5 min each, followed by three changes treatment with 95–100% alcohol and water for rehydration, 5 min each. Deparaffinated and rehydrated slides were then incubated for 40–60 min at 95–100 °C in citrate EDTA buffer (10 mM citric acid, 2 mM EDTA, 0.05% Tween 20, pH 6.2) for antigen retrieval and unmasking epitopes for antibody binding. In order to block non-specific labeling, samples were incubated with 5% serum in PBS-T (5 mL normal donkey serum, 0.5 mL 20% Triton X-100, 10 mL 10× PBS, 84.5 mL ddH2O) in a humidified chamber at RT for 1 h. Then, sections were incubated overnight at 4 °C with the blocking buffer containing one of the following primary antibodies: mouse monoclonal anti-TRPV1, VR-1 (sc-398417, Santa Cruz Biotechnology Inc, Dallas, USA) for TRPV1 immunostaining [59], chicken polyclonal anti-glial fibrillary acid protein, GFAP (ab4674, Abcam, Toronto, ON, Canada) for astrocytes immunostaining [60], mouse monoclonal anti-ionized calcium binding adapter molecule, Iba-1 (MA5-27726, TermoFisher scientific, Rockford, AZ, USA) for microglia immunostaining [61], rabbit monoclonal anti-calcitonin gene related peptide IgG, CGRP (sp-17, Abcam, Toronto, ON, Canada) for peptide sensory fibers immunostaining [62], fluorescein labeled GSL I isolectin B4, IB4+ (FL-1201, Vector laboratories, Burlingame, CA, USA) for non-peptide sensory fibers immunostaining [63], polyclonal Goat anti-IL1-β IgG (PA5-46956, Polyclonal Goat IgG, TermoFisher scientific, Rockford, AZ, USA) to identify IL-1β [64] and a polyclonal rabbit antiserum for B1R (Biotechnology Research Institute, Montreal, QC, Canada) for kinin B1R immunostaining [65,66]. On the next day, antibody solution was removed, and samples were washed three times with buffer (1 mM PBS) for 5 min each followed by incubation with secondary antibodies (diluted in 1mM PBS solution 1:200) at RT for 1 h. The secondary antibodies used in this study are Alexa Fluor 555 (Donkey anti-mouse IgG (A31570, Invitrogen, Montreal, QC, Canada), Alexa Fluor 633 (Donkey anti-goat IgG (A-21082, Invitrogen), Alexa Fluor 555 (Goat anti-chicken IgY (A-21437, Invitrogen) and Alexa Fluor 488 (Donkey anti-rabbit IgG (A21206, Invitrogen). Thereafter, the secondary antibody solution was removed; slides were washed three times in wash solution, then dried and mounted using an antifade reagent. Images were obtained with Olympus IX2 inverted microscope (DSU; Olympus, Tokyo, Japan). The examination was limited to the dorsal horn under confocal microscope (Leica Confocal microscope, Richmond Hill, ON, Canada).

For enzymatic detection of B1R, immunohistochemistry HRP staining protocol was used [67]. Samples were processed as described before except an additional peroxidase-blocking step was added to prevent non-specific background resulting from conjugated HRP use. The day after the incubation of the rabbit B1R antiserum, slides proceeded as follows: sections were incubated in peroxidase blocking solution (0.3% H_2_O_2_ in PBS) for 10 min at RT; then, biotin-conjugated, goat anti-rabbit secondary antibody was applied to the slides and incubated for 2 h at RT followed by an incubation in Streptavidin-HRP for 1 h (for detection of biotinylated antibodies). For immunohistochemical staining, a DAB (3,3′-Diaminobenzidine)–peroxidase substrate solution was made as follows: 250 µL of 1% DAB, 250 µL of 0.3% hydrogen peroxide mixed in 5 mL of 0.05% PBS, the pH of the final solution being buffered to 7.2. Slides were treated for 10 min. Rinsing of slides in 1mM PBS wash solution followed each step. Finally, slides were passed through xylene and ethanol 95 and 100% (2 times), and then mounted with DPX medium for further visualization.

### 4.11. Densitometric Analysis

Densitometric analysis of B1R distribution was made in five rats (*n* = 5) randomly selected from each group as described elsewhere [68]. Tissue sections of the lumbar spinal cord were analyzed by using computer-based MCID-M1 analysis software, version 4.20 Rev.1.0 (Imaging Research, St. Catharines, ON, Canada). Images of the targeted sections were captured with Dage-MTI CCD72 monochromatic camera (Michigan City, IN, USA) by using 2.5 objectives, and were displayed in 8-bit mono format for further analysis. Step-by-step sections were scanned and analyzed on the full-scale image display (256 × 256 pixels). The digitalized signal of the selected regions was analyzed in a range of 0–255 pixels and the optical density (O.D.) values were displayed in a range of 0.1–1.0. Each value was calculated as a mean of five sections per rat (*n* = 5), so that we analyzed 25 sections per group.

### 4.12. Statistical Analysis of Data

Statistical analysis was carried out in accordance with the recommendations of experimental data analysis in pharmacology [69] using Prism^TM^ version 5.0 GraphPad Software (La Jolla, CA, USA) and Excel 2016 Software (Microsoft). Data were expressed as means ± S.E.M. obtained from *n* rats. Statistical significance between groups (control/treated and vehicle-treated/antagonist-treated) was determined by using a Student’s *t*-test for unpaired samples. One-way or two-way ANOVA followed by the Bonferroni test were used for multiple comparisons. *p* values of ≤ 0.05 were considered statistically significant.

## 5. Conclusions

While B1R and TRPV1 contributed to thermal hyperalgesia, they play a distinctive role in allodynia associated with NP. Whereas a direct interaction between B1R and TRPV1 is possible in this model of NP, astrocytes surrounding nociceptors allow an indirect and close interaction between B1R and TRPV1, which are both colocalized on these two structures. Indeed, the pharmacological approach using B1R and TRPV1 antagonists suggests a reciprocal regulatory mechanism between B1R and TRPV1 expression as B1R antagonism suppressed TRPV1 upregulation while TRPV1 blockade caused the upregulation of IL-1β and B1R after PSNL. IL-1β may act as an astrocyte messenger to enhance B1R expression. This study raises the possibility that TRPV1 antagonism may exert a counterproductive effect in the treatment of NP by enhancing simultaneously the expression and function of IL-1β and B1R.

## Figures and Tables

**Table 1:** TNF-α and IL-1β mRNA levels in the Spinal Cord and dorsal root ganglion (DRG) after treatment with B1R antagonist (SSR240612) and TRPV1 antagonist (SB366791).

Side	Treatment	Spinal Cord (Fold Expression)		DRG (Fold Expression)	
		TNF-α	IL-1β	TNF-α	IL-1β
SSR240612					
*Contralateral*	Control	1	1	1	1
	Vehicle	3.2 ± 0.1 *	2.5 ± 0.2	0.6 ± 0.3	0.5 ± 0.3
	SSR240612	5.5 ± 0.4 *^+^	4.7 ± 0.3 *^+^	0.9 ± 0.1	0.9 ± 0.2
*Ipsilateral*	Vehicle	3.8 ± 0.1 *	2.3 ± 0.1	3.9 ± 0.2 *	0.2 ± 0.5
	SSR240612	5.1 ± 0.4 *^+^	5.0 ± 0.3 *^+^	3.6 ± 0.2 *	0.4 ± 0.3
SB366791					
*Contralateral*	Control	1	1	1	1
	Vehicle	3.1 ± 0.1 *	2.0 ± 0.3	0.4 ± 0.2	0.2 ± 0.1
	SB366791	3.3 ± 0.7 *	5.0 ± 0.3 *^+^	0.1 ± 0.1	0.4 ± 0.3
*Ipsilateral*	Vehicle	4.0 ± 0.2 *	1.8 ± 0.2	3.1 ± 0.3 *	0.3 ± 0.2
	SB366791	4.4 ± 0.3 *	4.0 ± 0.3 *^+^	4.4 ± 0.4 *	0.3 ± 0.3

Values are fold-change ± SEM of control (*n* = 7), vehicle-treated (*n* = 7), SB366791-treated (*n* = 8) and SSR240612-treated (*n* = 8) groups. Statistical significance is indicated by * *p* < 0.05, (F = 4.74, *p* < 0.001) vs. control and ^+^ *p* < 0.05, (F = 4.74, *p* = 0.02) vs. vehicle.
